# Comprehensive catalog of gut microbial genomes in Asian elephants: insights from shotgun metagenomics

**DOI:** 10.1186/s42523-026-00533-0

**Published:** 2026-03-03

**Authors:** Xingwei Shi, Fei Chen, Min Dai, Yongjing Tang, Jishan Wang, Yuheng Lin, Minhui Shi, Tianming Lan, Huan Liu, Xin Jin, Liang Xiao, Karsten Kristiansen, Xiaoping Li

**Affiliations:** 1 BGI Research, Wuhan, 430074 P. R. China; 2 College of Life Sciences, University of Chinese Academy of Sciences, Beijing, 100049 P.R. China; 3 Institute of International Rivers and Eco-Security, Yunnan University, Kunming, 650500 P.R. China; 4 Asian Elephant Research Center of National Forestry and Grassland Administration, Kunming, 650031 P.R. China; 5 Laboratory of Integrative Biomedicine, Department of Biology, University of Copenhagen, Copenhagen, 2100 Denmark; 6 Shenzhen Engineering Laboratory of Detection and Intervention of Human Intestinal Microbiome, BGI- Shenzhen, Shenzhen, 518083 P.R. China; 7 BGI Research, Shenzhen, Guangdong, 518083 P.R. China; 8 Shenzhen Key Laboratory of Environmental Microbial Genomics and Application, BGI-Shenzhen, Shenzhen, 518083 China

**Keywords:** Asian elephant, Gut microbiota, Metagenome, Migration, Lifestyle

## Abstract

**Background:**

The gut microbiota plays a crucial role in metabolism, immune regulation, and ecological adaptation of mammals. Although significant advancements have been made in shotgun metagenomic sequencing and the emergence of algorithms for generation of metagenome-assembled genomes (MAGs), a comprehensive investigation of the gut microbiota at the species level of wild mammals, among these the Asian elephant (*Elephas maximus*), is still lacking.

**Results:**

Here, based on a total of 82 fresh fecal samples collected from Asian elephants residing in distinct regions of the Yunnan Province, we established a comprehensive genome catalog containing 1421 species-level genome bins (SGBs) and a gene catalog comprising 44,596,628 non-redundant genes covering the gut microbiota composition of representative Asian elephant populations. At the species level, 1368 bacteria and 53 archaea were identified, and more than 93% of the SGBs remained unclassified, indicating that there are a large number of potential new species in the Asian elephant gut microbiota. At the functional level, carbohydrate hydrolases, biosynthetic gene clusters, and metabolic pathways dominated the gut microbiome of Asian elephants. Lifestyle and migration affected the composition and functional potential of the gut microbiota of Asian elephants. A northward migration was accompanied by an increase in gut microbiota diversity, an increase in the abundance of the phylum Bacteroidota, and a decrease in the presence of potentially pathogenic genera. In contrast, a southward migration of elephant herds was accompanied by exposure to unfavorable environments, with changes in gut microbiota including increased xenobiotic degradation and metabolic capacity.

**Conclusions:**

We constructed comprehensive catalogs of gut microbial genes and genomes representative for Asian elephant populations, providing a valuable data resource for future research. Our study elucidates migration and lifestyle may modulate the composition and functionality of the gut microbiota in Asian elephants, offering critical insights for monitoring their health and enhancing conservation strategies for wild populations.

**Supplementary Information:**

The online version contains supplementary material available at 10.1186/s42523-026-00533-0.

## Introduction

The gut microbiota is essential for host development, immune regulation, and other physiological processes [1]. While the human microbiota has been extensively studied [2–6], studies on the animal microbiota have primarily focused on rodents and domestic animals [7–12], and only recently has the microbiota of wild animals received attention [13]. The gut microbiome of herbivorous mammals’ harbors genes associated with the degradation of structural polysaccharides, thereby serving as a primary catalyst for plant cell wall degradation [14–16]. In addition, the intestinal microbiota exhibits notable flexibility, undergoing changes in composition and function in response to variations in the external environment [17]. Hence, the microbiota can serve as a valuable indicator for monitoring the nutritional status of wildlife. The investigation of the gut microbiota in wild animals has aided the advancement of wildlife conservation. The Asian elephant (*Elephas maximus*) is the largest terrestrial herbivorous mammal in Asia, and their gut microbiota plays a crucial role in the degradation of cellulose within its diet [18]. In recent years, previous research on the Asian elephant gut microbiota has predominantly focused on taxonomic classification, cellulose-degrading microbial communities, and alterations caused by environmental changes [17–21]. These studies have contributed to our understanding of the intricate community structure of the Asian elephant gut microbiota and have underscored its significance in relation to the growth and development of these wild animals. The advancements in shotgun metagenomics sequencing technologies, coupled with the development of the algorithms for construction of metagenome-assembled genomes (MAGs) [22, 23], have enabled a detailed characterization of microbial community composition at the species level. Here, taking advantage of an Asian elephant migration event in the Yunnan province in 2021 [24, 25], we were able to collect samples from two herds of Asian elephants who had undertaken migrations from Mengyang to the Kunming area and the Xishuangbanna Tropical Botanical Garden (XTBG), enabling analyses of the impact of migration on the gut microbiota. Thus, a total of 82 fresh fecal samples were collected from five distinct regions of the Yunnan province, Kunming, Mengyang, Jingne, the Wild Elephant Valley (WEV), and the XTBG. Based on these samples, we constructed a comprehensive gut microbial genome catalog representing the different Asian elephant populations. We examined the influence of migration and lifestyle on gut microbial composition and function, which we envisaged will be of value for future projects on the conservation of the Asian elephant.

## Material and methods

### Sample collection

In total 82 fresh samples from Asian elephants (*Elephas maximus*) were systematically collected in June 2021 across five regions in the Yunnan Province, China: Kunming, Xishuangbanna Tropical Botanical Garden (XTBG), Jingne, Mengyang, and Wild Elephant Valley (WEV). 9 Samples from WEV came from captive elephants rescued from the wild population or their offsprings, while samples from the other regions were from wild elephants. Sampling targeted two distinct migratory groups originating from Mengyang in March 2020 [24, 25]: (1) the Kunming group comprising 27 samples collected from a herd migrating northward toward Kunming, and (2) the XTBG group comprising 33 samples obtained from a population moving southward toward Xishuangbanna Tropical Botanical Garden. Both groups had initiated the migration from their natural habitat more than one year prior to sampling. For comparison, we also collected samples from wild, non-migrating populations in Jingne comprising 11 samples and Mengyang comprising 2 samples (the geographic distribution is detailed in Supplementary Table 1A and Supplementary Fig. 1).

All samples were collected in sterile containers using standardized protocols. Using the MGIEasy Fecal Sample Collection Kit, the internal core of a fresh fecal sample was collected using a sterile spatula and transferred to a sampling tube. All fecal samples were stored at −80 °C until they were transported on dry ice to the China National GeneBank (CNGB) in Shenzhen for further analysis.

### DNA extraction, sequencing, and quality control

The MagPure Stool DNA KF Kit B (MAGEN, Guangzhou, China) was used for DNA extraction according to the manufactory’s instruction. The sample transfer and other procedures were conducted within a sterilized ultra-clean workbench, adhering to the appropriate protocol [26, 27]. The MGIEasy Universal DNA Library Prep Set (MGI-Shenzhen, China) was used to construct DNA sequencing library with an insert size of 350 bp. After library construction, qualified samples were sequenced on the BGI DNBSEQ T1&T5 sequencer using 150 bp paired-end reads. A total of 1.51 Tb of raw reads were obtained by sequencing the 82 fecal samples. Using the ‘–length_required 70’ option of fastp v0.20.1 [28] for quality control, reads with a length of less than 70 bp were filtered out. We obtained 18.23 ± 4.26 Gb clean reads per sample. The Asian elephant genome data (GCA_014332765.1) was indexed using Bowtie2 v2.4.2 [29], and host-contaminating reads were removed by alignment. Finally, high-quality reads of 18.21 ± 4.26 Gb per sample were obtained for assembly.

### Metagenomic assembly and binning

We assembled high-quality reads in each sample individually using MEGAHIT v1.2.9 [30], filtered contigs below 200 using the ‘–min-contig-len 200’ option. We adopted the multi-coverage binning strategy and used MetaBAT2 v2.15 [31] to obtain MAGs based on the tetranucleotide frequency and contig abundance correlation of ten samples. MAGs were evaluated for completeness and contamination using CheckM [32] v1.1.3’s lineage_wf mode, and the quality score (QS, defined as completeness – (5×contamination)) was calculated. A total of 6313 effective MAGs were obtained (completeness ≥ 50%, contamination < 10% and QS ≥ 50%), including 2040 high quality MAGs (completeness > 90% and contamination < 5%). We used GUNC v1.0.5 [33] to perform the quality assessment on the effective MAGs with the options “–sensitive –use_species_level” to filter genomes that might contain chimeras according to “pass.GUNC” in the output file. The ribosomal RNA (rRNA) was searched using Barrnap v0.9 (https://github.com/tseemann/barrnap) with the option “–quiet”. The transfer RNAs (tRNAs) were identified using tRNAscan-SE v2.0.5 (https://github.com/UCSC-LoweLab/tRNAscan-SE), applying the option “−A” for archaeal species and “−B” for bacterial species, according to the Genome Taxonomy Database Toolkit (GTDB-Tk) [34] v2.3.2 annotations.

### Clustering MAGs into species-level genome bins (SGBs)

The 6313 effective MAGs were clustered into strain-level genomes (average nucleotide identity (ANI) ≥ 99%) using dRep v2.6.2 [35] with options: ‘–MASH_sketch 10,000 –S_algorithm ANImf –P_ani 0.95 –S_ani 0.99 –cov_thresh 0.3’. We selected the representative genome with the highest genome quality score: completeness − 5 × contamination + 0.5log (N50) for each strain cluster. The representative strain genomes were further clustered into species-level genomes (ANI ≥ 95%) using the same options as above except that “–P_ani 0.90 –S_ani 0.95”, was used for selecting the representative genomes with the highest genome quality scores.

### Mapping rate

We mapped high-quality reads to the 1421 SGBs of elephant gut microbes using Kraken2 v2.1.1 [36], and counted the mapping rate of each sample. In addition, we counted the mapping rates to the Genome Taxonomy Database (GTDB) r214 and NCBI Reference Sequence Database (RefSeq) (20210517). The GTDB includes 85,205 species from bacteria and archaea, and the RefSeq standard database includes sequences from archaea, bacteria, viruses, plasmids, humans, and UniVec Core.

### Species annotation and construction of a phylogenetic tree

Species annotation was performed for the 1421 SGBs using GTDB-Tk v2.3.2 [34] based on the GTDB r214. Marker single-copy genes were identified (53 for archaea and 120 for bacteria) and the number of marker genes in each SGB was counted. We used phylophlan v3.0.67 [37] to construct the phylogenetic tree of the 1421 SGBs based on 400 universal marker genes, with the options “-d phylophlan –diversity high –accurate –min_num_markers 100”. The final phylogenetic tree including 1406 SGBs was visualized using iTol v6 [38].

### Calculation of the relative abundance of SGBs

We used Bowtie2 v2.4.2 [29] with options ‘–end-to-end –sensitive -k 2’ to align high-quality reads from 82 samples to the genome catalog containing 1421 SGBs. Then, unique alignments that met the following two criteria were retained: 1) both ends of the read aligned to the same contig; 2) the alignment identity was no less than 95%. To reduce false-positive results, at least 10 paired-end reads were required to support the presence of a given species in each sample, and the relative abundance of SGBs was calculated as previously described [26].

### Microbial diversity, principal coordinate analysis (PCoA) and permutational multivariate analysis of variance analyses (PERMANOVA)

Based on the relative abundances of SGBs in each sample, the α diversity of the Asian elephant gut microbiota in each region was estimated using the R vegan package, including the richness and Shannon index. Bray-Curtis dissimilarities between each sample were calculated using “vegdist (method = ‘bray’)” from the R Vegan package and PCoA was performed using the “cmdscale” function. PERMANOVA analysis was performed using the “adonis2” function in the R Vegan package based on the Bray-Curtis dissimilarities to calculate the coefficients of determination (R^2^) for each effect factor on the gut microbiota of Asian elephants.

### Gene prediction and profile

Based on the assembled contigs, we used prodigal v2.6.3 [39] with the option ‘−p meta’ for gene prediction, whereby a total of 216,081,398 genes were obtained. All genes were clustered according to 95% average nucleotide identity and 80% coverage, and finally 44,596,628 non-redundant genes were obtained. The software used in the clustering process was MMseqs2 v13.45111 [40], and the options were “-c 0.8 –kmer-per-seq 80 –min-seq-id 0.95”. We used Salmon v0.8.2 [41] to construct the index of the non-redundant gene catalog, profiled the genes in each sample, and finally obtained the transcript per million (TPM) value as the relative abundance of the genes.

### Functional annotation

We functionally annotated the protein sequences encoded by the non-redundant genes using eggnog-mapper [42] and eggnog Orthologous Groups database v5.0.2 [43]. The eggnog database integrates functional annotation databases from multiple sources, including the Kyoto Encyclopedia of Genes and Genomes (KEGG) Ortholog Database, the Carbohydrate Active Enzyme (CAZyme) Database, and the Class of Ortholog Groups (COGs). Virulence factors (VFs) were identified by aligning protein sequences of non-redundant genes to the virulence factor database (VFDB) using blastp v2.13.0+ with options “E-value < 1e − 5, query match length > 50% and identity > 60%”. We only aligned core datasets containing representative genes associated with experimentally verified VFs. Antibiotic resistance genes (ARGs) were identified by aligning protein sequences of non-redundant genes with the Comprehensive Antibiotic Research Database (CARD) v3.2.3 [44] using RGI v5.2.0 with parameters “identity > 80%”. Annotation of CAZymes in the protein sequences was performed using dbCAN v12 [45], a collection of Hidden Markov Model (HMM) profiles built based on the CAZyme database. Hits were filtered for a minimum coverage of 35% and an E-value less than 1e − 18 [46].

### Prediction of biosynthetic gene clusters (BGCs)

We predicted BGCs from our recalled SGBs using antiSMASH v6.0.0 [47] based on the profile Hidden Markov Models (pHMMs) from PFAM, TIGRFAMs or custom models. The predicted BGCs were compared with the Minimum Information about a Biosynthetic Gene Cluster (MiBGC) database to determine whether they were BGCs with known functions. We used BiG-SCAPE v1.1.5 [48] to assign gene cluster families (GCFs) of the obtained BGCs.

### Rarefaction curve

We constructed rarefaction curves of the sample size and the number of SGBs to assess whether the sample size was sufficient. The 1421 SGBs’ curves were created by randomly re-sampling the pool of 82 samples 10 times with 5 sampling intervals, then all SGBs and non-singleton SGBs were plotted using the geom_smooth functions from the R ggplot2 package. We constructed rarefaction curves of sample size and number of all genes and core genes (present in more than 50% of the samples) to assess the adequacy of the sample size. The curves were created from the gene numbers obtained by randomly resampling 82 samples 10 times at 10 sampling intervals, and then plotted using the geom_smooth function in the R ggplot2 package.

### Difference analysis

In terms of species composition, we first used the non-parametric factorial Kruskal-Wallis rank-sum test to detect species that differed significantly in abundance between different groups in multiple groups of samples, and these species were analyzed by grouped Wilcoxon rank-sum test. Finally, linear discriminant analysis (LDA) was used to reduce the dimensionality of the data and evaluate the influence of species that differed significantly in abundance (LDA score). At the functional level, we used the Wilcoxon rank-sum test to analyze differences between groups, using *p* < 0.05 as the threshold for significant difference between the two groups.

## Results

### Construction of microbial genomes from gut bacteria of the Asian elephant

We performed metagenomic sequencing of 82 fecal samples from Asian elephants from five different regions (Supplementary Fig. 1 and Supplementary Table 1A), generating more than 1.51 Tb of raw sequencing data (Supplementary Table 1B). After quality control of reads, we used Metabat2 [31] for multi-coverage binning, using the contigs from single-samples assemblies created with MEGAHIT [30]. This process yielded 8482 high- and medium-quality MAGs (≥50% completeness and < 10% contamination) following the “Minimum information about a metagenome-assembled genome (MIMAG)” standard. Of these, 6313 effective MAGs matched a QS ≥ 50%. The median of the genome size was 2.27 Mb (interquartile range, IQR = 1.72–2.72 Mb) and the median size of N50 was 10.36 Kb (IQR = 5.30–24.84 Kb). Among the effective MAGs, 2040 (32.31%) were high quality (completeness > 90%, contamination < 5%), and 4273 (67.69%) were medium quality (completeness ≥ 50%, contamination < 10%) (Supplementary Fig. 2). 85.82% (5,418) of the effective MAGs contained no chimeras, and 64.55% (4,075) of the effective MAGs included at least one 5S rRNA gene, 16S rRNA gene or 23S rRNA gene, and at least 18 tRNA genes (Supplementary Table 2).

**Fig. 1:**
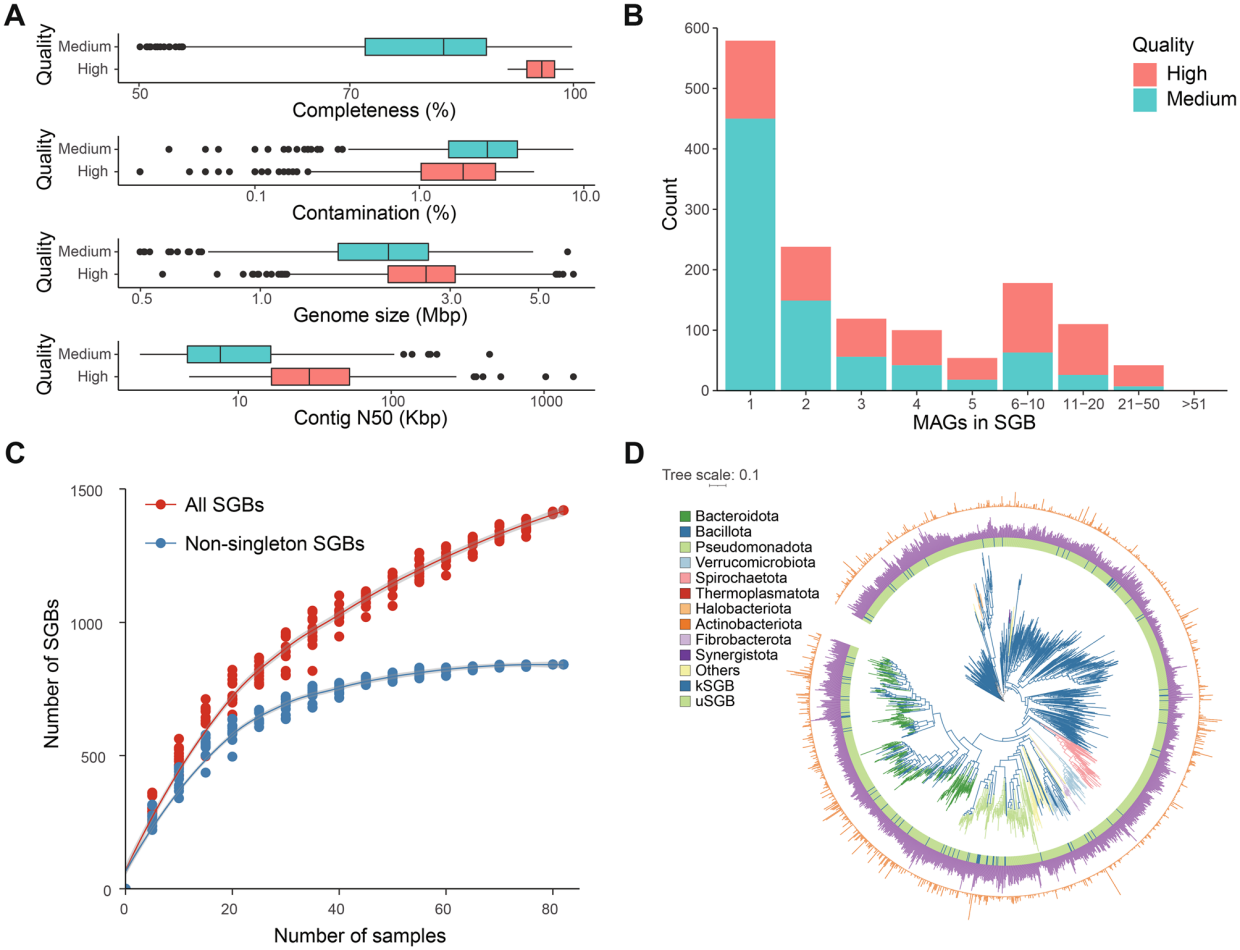
Reference genome catalog of gut microbes in Asian elephants. (**A**) quality assessment of representative genomes is depicted, with completeness, contamination, genome size, and N50 shown from top to bottom. High-quality genomes are highlighted in red boxes while medium-quality genomes are represented by blue boxes. (**B**) the distribution of the number of MAGs in SGBs. (**C**) rarefaction curve demonstrating species diversity estimates for the 1421 SGBs obtained from all 82 samples. Curves were generated by randomly resampling the pool of 82 samples ten times at five sampling intervals for both all SGBs (red line) and non-singleton SGBs (blue line). (**D**) phylogenetic analysis performed using phylophlan v3.0.67 on the 1421 SGBs based on 400 universal marker genes resulting in a phylogenetic tree comprising 1406 filtered SGBs with at least 100 marker genes each. Branches in the phylogenetic tree are color-coded according to phylum classification, while the three rings indicate known/unknown species, genome size, and number of MAGs per species

We clustered the 6313 effective MAGs into 1421 SGBs with 95% ANI, including 610 (42.93%) high-quality SGBs (Supplementary Table 3). Among the high-quality SGBs, the genome size distribution ranged from 0.57 to 6.12 Mb, with the average value of 2.63 Mb (Fig. 1A). The average values of N50 and N90 were 47.58 Kb and 13.00 Kb, respectively (Fig. 1A). Among the SGBs, 842 (59.25%) of the SGBs were supported by at least two MAGs, while 579 (40.75%) of the SGBs were only supported by a single MAG (Fig. 1B), indicating that both prevalent and rare species were assembled. Rarefaction curve analysis demonstrated that non-singleton SGBs reached a plateau, indicating a stable level of diversity. However, the overall species richness had not yet reached saturation (Fig. 1C), implying the potential for discovering additional species, particularly rare ones, as sampling efforts expand. High-quality reads from each sample were successfully mapped to 1421 SGBs, achieving a mapping rate exceeding 60% (Supplementary Fig. 3A). This rate is significantly higher than those observed in RefSeq (17.73%) and GTDB r214 (47.35%) (Supplementary Fig. 3A). These results indicate that our genome catalog of Asian elephant gut microbes comprehensively captures the majority of abundant microbial species present in the gut of Asian elephants. The 1421 SGBs were taxonomically classified using GTDB-Tk [34]. Among these, 1368 were identified as Bacteria (Supplementary Fig. 3B) and 53 as Archaea (Supplementary Fig. 3C). Furthermore, 1420 SGBs were assigned to 23 phyla (3 for Archaea and 20 for Bacteria), 28 classes (3 for Archaea and 25 for Bacteria), and 76 known orders (3 for Archaea and 73 for Bacteria). Additionally, 1419 SGBs (99.86%) could be classified into 142 known families (3 for Archaea and 139 for Bacteria), while 1299 SGBs (91.41%) were classified into 360 known genera (7 for Archaea and 353 for Bacteria). At the species level, over 93.80% of the SGBs remained unclassified, suggesting a significant presence of potentially novel species within the gut microbiota of Asian elephants. Most bacterial genomes (85.60%) were classified into four major phyla: Bacillota_A (508), Bacteroidota (360), Bacillota (160), and Pseudomonadota (143). Several genomes with specific functional roles were also identified in our genome catalog. Notably, within the Fibrobacterota phylum, which plays a critical role in cellulose degradation in the gastrointestinal tracts of cattle and pigs, we discovered 11 previously unknown species from the gut microbiota of Asian elephants, representing a 7.43% increase in diversity relative to GTDB (Supplementary Fig. 3D and Supplementary Table 4). Moreover, archaeal genomes were classified into three phyla: Thermoplasmatota (27), Halobacteriota (22), and Methanobacteriota (4) (Supplementary Fig. 3C). All archaeal genomes belonged to three families: Methanomethylophilaceae, Methanocorpusculaceae, and Methanobacteriaceae. Importantly, 52 potentially novel archaeal species were identified, all of which are involved in methane production.

Based on the genome catalog of the Asian elephant gut microbiota, we functionally annotated all genes in the 1421 species and identified biosynthetic gene clusters (BGCs) that encode secondary metabolites crucial for microbial survival. We identified a total of 3793 BGCs from 1257 sequenced genomes using antiSMASH v6.0.0 [47]. Surprisingly, only 3.22% of these BGCs were annotated in the MiBGC database, suggesting a significant potential for discovering novel functional pathways within these genomes (Supplementary Table 5). The predicted production of secondary metabolites was further classified into seven superfamilies using BiG-SCAPE [48] v1.1.5. Among these, ribosomally synthesized and post-translationally modified peptides (RiPPs) showed the highest diversity, encompassing 1426 BGCs, which accounted for 37.60% of the total number of identified BGCs (Supplementary Fig. 3E). We found that most RiPPs were from Bacillota_A (Supplementary Fig. 3E). Non-ribosomal peptide synthetases (NRPS) ranked second, accounting for 32.80% of the total identified BGCs (Supplementary Fig. 3E). Annotation based on CARD revealed that 39 SGBs carried 131 ARGs (Supplementary Table 6). The main drug classes of these ARGs comprise fluoroquinolone antibiotics, penams, cephalosporins, phenicol antibiotics, and tetracycline antibiotics. *Escherichia coli* exhibited the highest abundance of ARGs, with a total of 46 ARGs, followed by *Klebsiella variicola* (21 ARGs) and *Klebsiella electrica* (13 ARGs). A previous study demonstrated that *K. variicola* has the potential to acquire and disseminate antimicrobial resistance genes in both clinical and environmental settings [49]. At the phylum level, the SGBs of Pseudomonadota harbored the highest abundance of ARGs, encompassing a total of 120 distinct ARGs. The genes were also annotated using the VFDB, resulting in the annotation of 17,275 VFs from 1380 SGBs. These VFs primarily encompassed immune modulation, adherence, motility, stress survival, and effector delivery systems (Supplementary Table 7). At the species level, *E. coli* harbors the highest number of virulence factors (350 VFs), followed by *Pseudomonas_E oleovorans_B* (242 VFs) and *K. variicola* (173 VFs). At the phylum level, SGBs belonging to Pseudomonadota carry the most diverse repertoire of virulence factors, encompassing 7856 VFs, followed by Bacillota_A (4,341 VFs) and Bacteroidota (2,765 VFs). An alignment of 1421 SGBs to the dbCAN v12 database [45] was performed using run_dbcan, and we found that all 1420 SGBs contained genes encoding enzymes involved in degradation of carbohydrates, mainly concentrated in the phyla Bacteroidota, Bacillota_A, and Verrucomicrobiota (Supplementary Table 8). The primary substrates of these CAZymes include xylan, beta-glucans, host glycans, starch, and sucrose. Among the identified genes, 3893 encode CAZymes involved in cellulose degradation, which are predominantly found in genera such as *Fibrobacter* and *Treponema_D*.

### A catalog of gut microbial genes in the Asian elephant

**Fig. 2:**
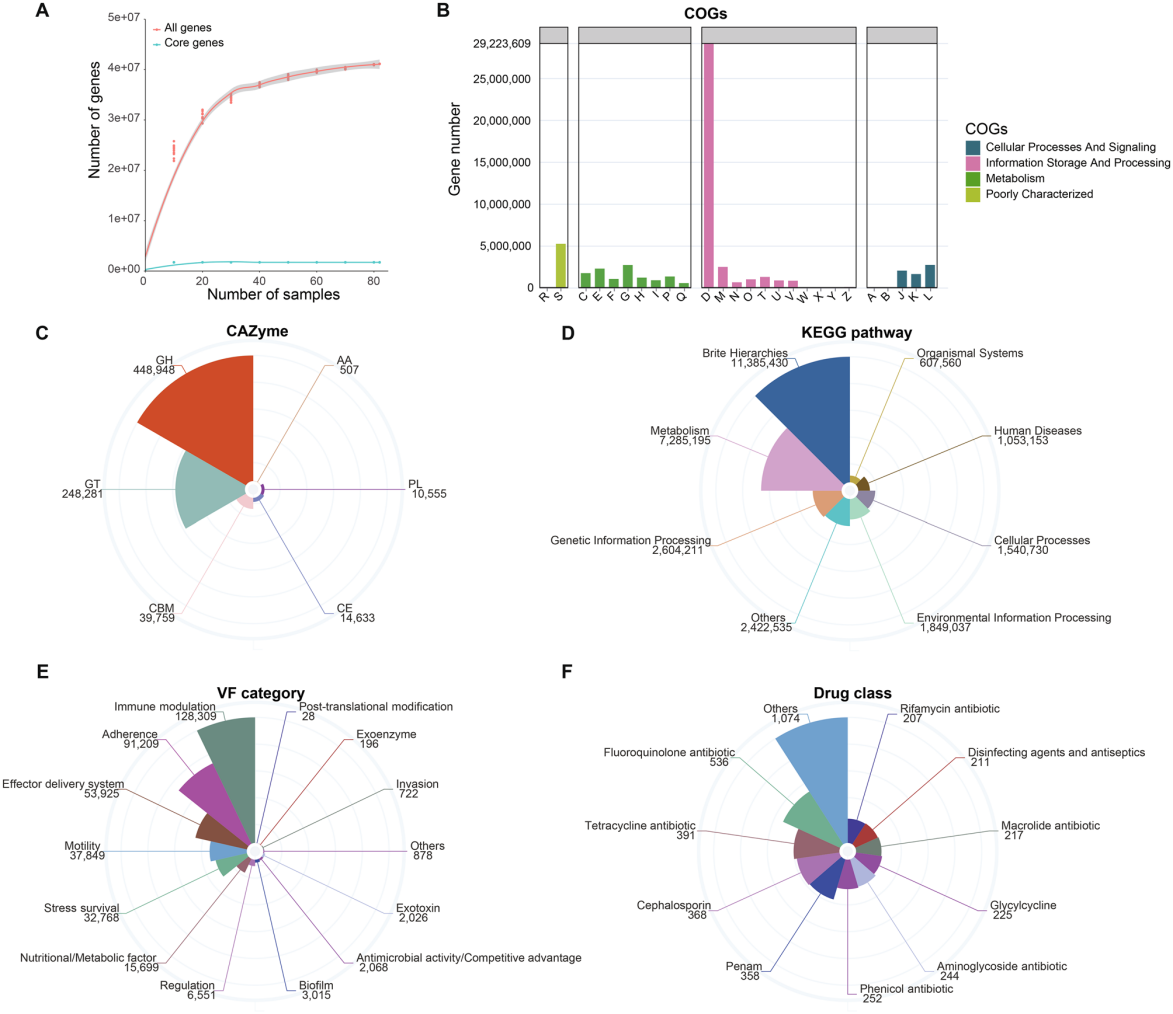
Gene catalog and functional characterization of the gut microbiome in Asian elephants. (**A**) rarefaction curves depicting the presence of 44,596,628 non-redundant genes obtained from all 82 samples (red line), as well as 1,727,995 non-redundant genes observed in more than 50% of the samples (green line). The curves were generated by randomly resampling a pool of 82 samples ten times at intervals of ten samplings. Gene distribution across COGs classification (**B**), CAZymes (**C**), KEGG pathways (**D**), VF categories (**E**), and drug classes (**F**)

Gene prediction was performed based on contigs assembled in each sample and all genes were clustered with 95% ANI. Finally, a non-redundant gene catalog of Asian elephant gut microbes containing 44,596,628 genes was generated. We found 1,727,995 non-redundant genes appearing in more than 50% of the samples, termed core genes. The rarefaction curve revealed that our sampling effort was insufficient to fully capture the gene richness of the elephant gut microbiome, as it did not reach a saturation plateau (Fig. 2A). However, it proved adequate for identifying core genes present in more than 50% of the samples (Fig. 2A). We investigated the functional characteristics of non-redundant gene catalog in Asian elephants. By employing the eggNOG-mapper [42], we compared the amino acid sequences encoded by these genes with the eggNOG database [43] and observed that 1.42% (632,925), 39.05% (17,413,034), and 64.38% (28,711,755) of the proteins were annotated as CAZymes, KEGG ortholog groups (KOs), and COGs, respectively (Fig. 2B-D). Most of these proteins were annotated as glycoside hydrolases (GHs, 448,948 (58.86%)) and glycosyltransferases (GTs, 248,281 (32.55%)) among the CAZymes. This was followed by carbohydrate-binding modules (CBMs, 39,759 (5.21%)). The remaining three classes included carbohydrate esterases (CEs, 14,633 (1.92%)), polysaccharide lyases (PLs, 10,555 (1.38%)), and proteins with auxiliary activities (AAs, 507 (0.07%)) (Fig. 2C). We identified numerous GHs associated with amylase degradation, including GH13, as well as GHs involved in cellulose degradation, such as GH3 and GH9, within the microbiome of Asian elephants. These GHs likely play a pivotal role in sustaining the energy demands of Asian elephants. In addition, the majority of the predicted proteins were annotated to Brite Hierarchies (39.60%) and Metabolism (25.34%), followed by Genetic Information Processing, Environmental Information Processing, and Cellular Processes (Fig. 2D). The metabolic pathways included carbohydrate metabolism, amino acid metabolism, energy metabolism, metabolism of cofactors and vitamins, and glycan biosynthesis and metabolism. The COG database annotation results revealed that a significant proportion of the predicted proteins are involved in cell cycle control, cell division, and chromosome partitioning functions (D). Metabolic functions were predominantly related to carbohydrate transport and metabolic activities (G) (Fig. 2B). These findings highlight the close interaction between the gut microbiota and the host as well as the presence of diverse metabolic pathways in gut bacteria in the Asian elephant compared to other animals [50]. By comparison with the VFDB, 375,243 open reading frames (ORFs) were identified as VFs. These ORFs belong to 390 VFs and 14 VF categories (Fig. 2E). Through alignment with CARD, we identified a total of 1235 ORFs encoding antibiotic resistance proteins, corresponding to 312 ARGs, including *rsmA*, *msbA*, *CRP*, *emrB*, and *acrD*. These ARGs encompass 30 distinct types of antibiotic resistance based on the classes of antibiotics they confer resistance to including fluoroquinolone, tetracycline, cephalosporin, penam and phenicol (Fig. 2F).

### Factors influencing composition and function of gut microbiota in Asian elephants

**Fig. 3:**
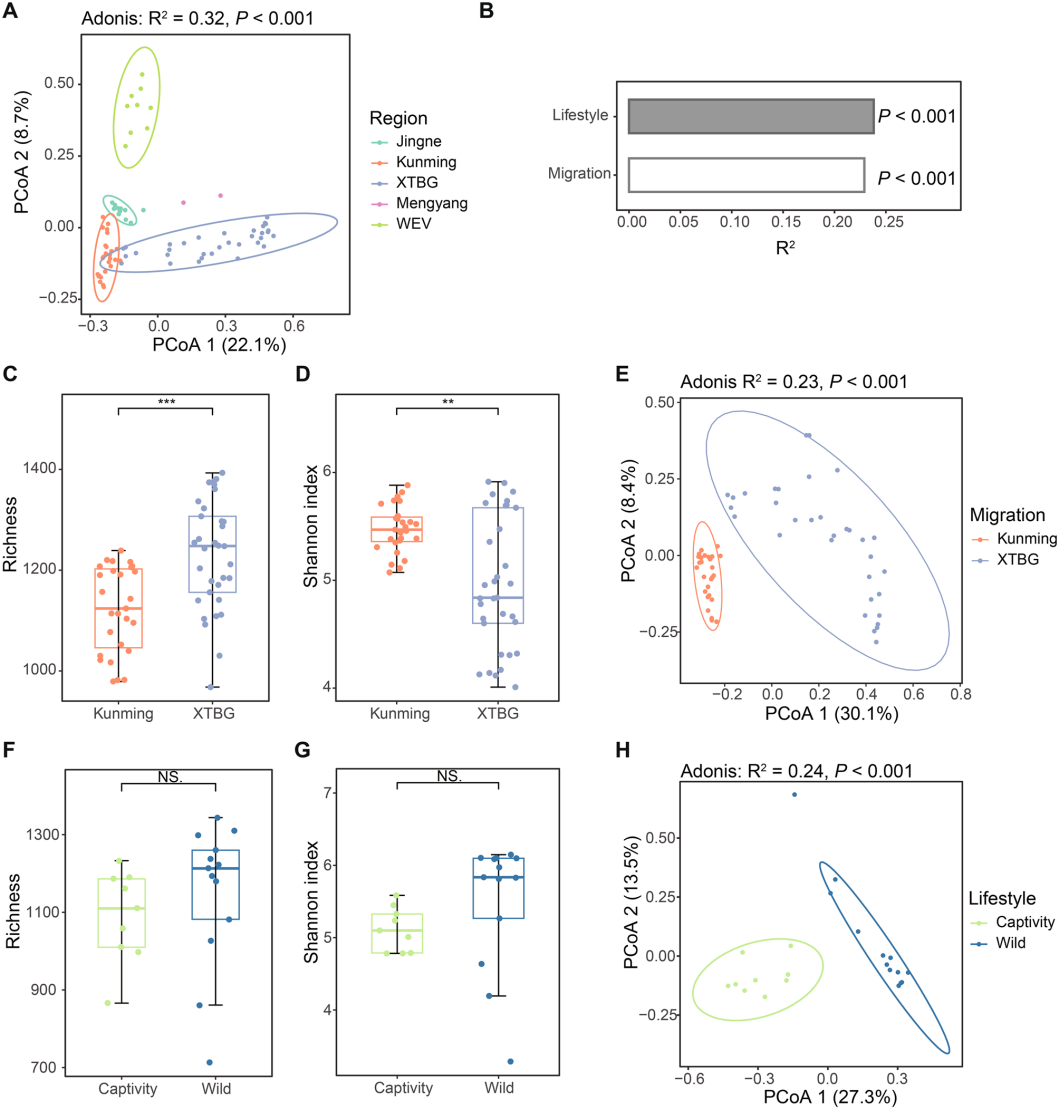
Factors influencing the gut microbiota composition in Asian elephants. (**A**) Principal coordinates analysis (PCoA) based on bray-Curtis dissimilarity, illustrating distinct clustering of gut microbial communities across five sampling regions. (**B**) PERMANOVA results quantifying the relative impact (R^2^ values) of different factors on gut microbiota composition. (**C-D**) α-diversity metrics (richness and Shannon index) comparing northern (Kunming) and southern (XTBG) migratory populations. (**E**) PCoA showing microbial community differentiation between northern and southern migratory groups. (**F-G**) α-diversity comparison between captive and wild populations. (**H**) PCoA demonstrating gut microbiota segregation between captive and wild elephants

We investigated to what extent the gut microbiota differed between the Asian elephants from the different groups. Based on the SGB profiles of all samples (Supplementary Table 9), our Principal Coordinates Analysis (PCoA) revealed that the intestinal microbiota of elephants could be clustered according to the sampled regions presumably reflecting different factors including lifestyle and migration routes (Fig. 3A).

We performed a PERMANOVA analysis to examine to what extent differences in the gut microbiota could be explained by lifestyle and migration. The result indicated that migration and lifestyle both affected the composition of the gut microbiota, migration (*p* < 0.001, R^2^ = 0.23), and lifestyle (*p* < 0.001, R^2^ = 0.24) (Fig. 3B).

### Effects of migration on gut microbial composition and function in Asian elephants

**Fig. 4:**
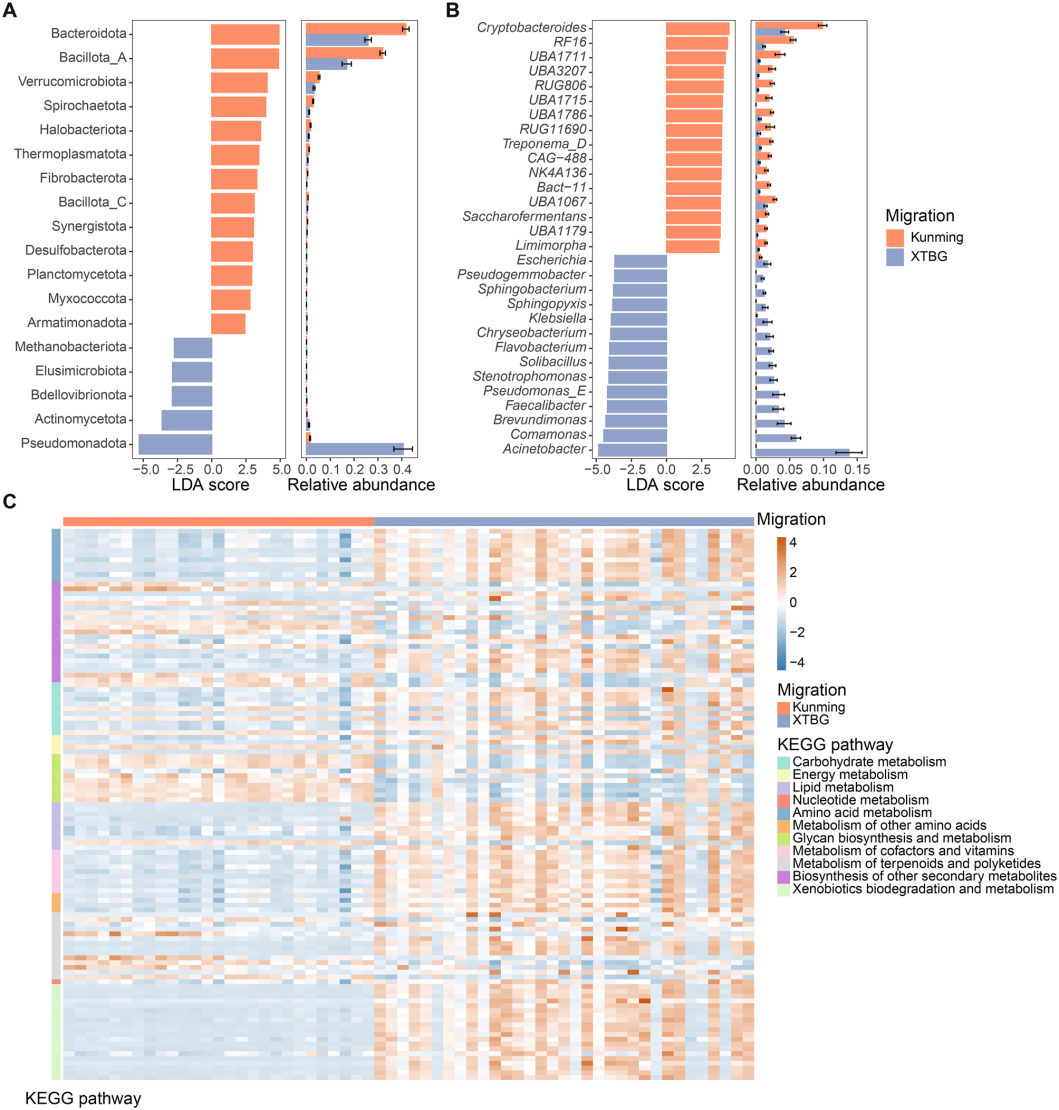
Effects of migration on gut microbial composition and function in Asian elephants. Comparative analysis of the species composition at the phylum (**A**) and genus level (**B**) between Kunming and XTBG. The left bar graph shows the LDA score, where the color indicates the direction of enrichment, while the right bar graph represents the relative abundance of the species. (**C**) differential enrichment analysis of KEGG pathway between the two groups

In order to investigate how the gut microbiota potentially could be influenced by migration, we compared Asian elephants migrating to the Kunming (north-migratory) to XTBG (south-migratory). We observed differences in richness and Shannon index between the north-migratory individuals and the south-migratory individuals (Fig. 3C-D). The PCoA analysis revealed a distinct separation between individuals following the northward and southward migrations direction (Fig. 3E). The alterations of microbial communities were examined using Linear Discriminant Analysis Effect Size (LEfSe) to reveal significant differences in species abundance between north-migratory and south-migratory individuals. The analyses showed that Bacteroidota and Bacillota_A were the most significantly enriched phyla in north-migratory Asian elephants (Fig. 4A). The phylum Pseudomondota differed significantly in abundance between the two groups and was mainly enriched in the south-migrating Asian elephants (Fig. 4A). At the genus level, *Cryptobacteroides*, *RF16*, and *UBA1711* were enriched in north-migratory Asian elephants, while *Acinetobacter*, *Comamonas*, and *Brevundimonas* were enriched in south-migratory Asian elephants (Fig. 4B). We observed a higher enrichment of CAZymes in the gut microbiota of north-migratory Asian elephants compared to south-migratory individuals. Specifically, this included 36 glycoside hydrolases (GHs), 17 glycosyltransferases (GTs), 5 carbohydrate-binding modules (CBMs), and 4 polysaccharide lyases (PLs) (Supplementary Fig. 4). GH families associated with cellulose degradation, such as GH3, GH5, and GH9, were more abundant in the gut microbiota of north-migratory Asian elephants than in south-migratory ones. Additionally, CBM families linked to hemicellulose (CBM42), carbohydrate (CBM48), and α-glucose degradation (CBM6) were enriched in the gut microbiota of north-migratory Asian elephants. Notably, PLs were exclusively enriched in the gut microbiota of north-migratory Asian elephants. For the CAZymes, those enriched in the gut microbiota of south-migratory Asian elephants primarily include glycosyltransferases (GT), such as GT51, GT16, GT56, and GT25, which are associated with polysaccharide synthesis and pathogen virulence production [51].

In the KEGG pathway analysis, the pathway of glycan biosynthesis and metabolism was enriched in the gut microbiota of north-migratory Asian elephants (Fig. 4C), and this result was consistent with the analyses of CAZymes. Noticeably, the pathway of xenobiotics biodegradation and metabolism was enriched in the gut microbiota of south-migratory Asian elephants (Fig. 4C). Furthermore, we found that VFs were enriched in south-migratory Asian elephants (Supplementary Fig. 5A). Finally, we found that more antibiotic resistance genes were enriched in south-migratory Asian elephants (Supplementary Fig. 5B). These results suggest that different migration routes and environmental factors significantly impacted the composition and function of the gut microbiota.

### Effects of captivity on gut microbial composition and function in Asian elephants

**Fig. 5:**
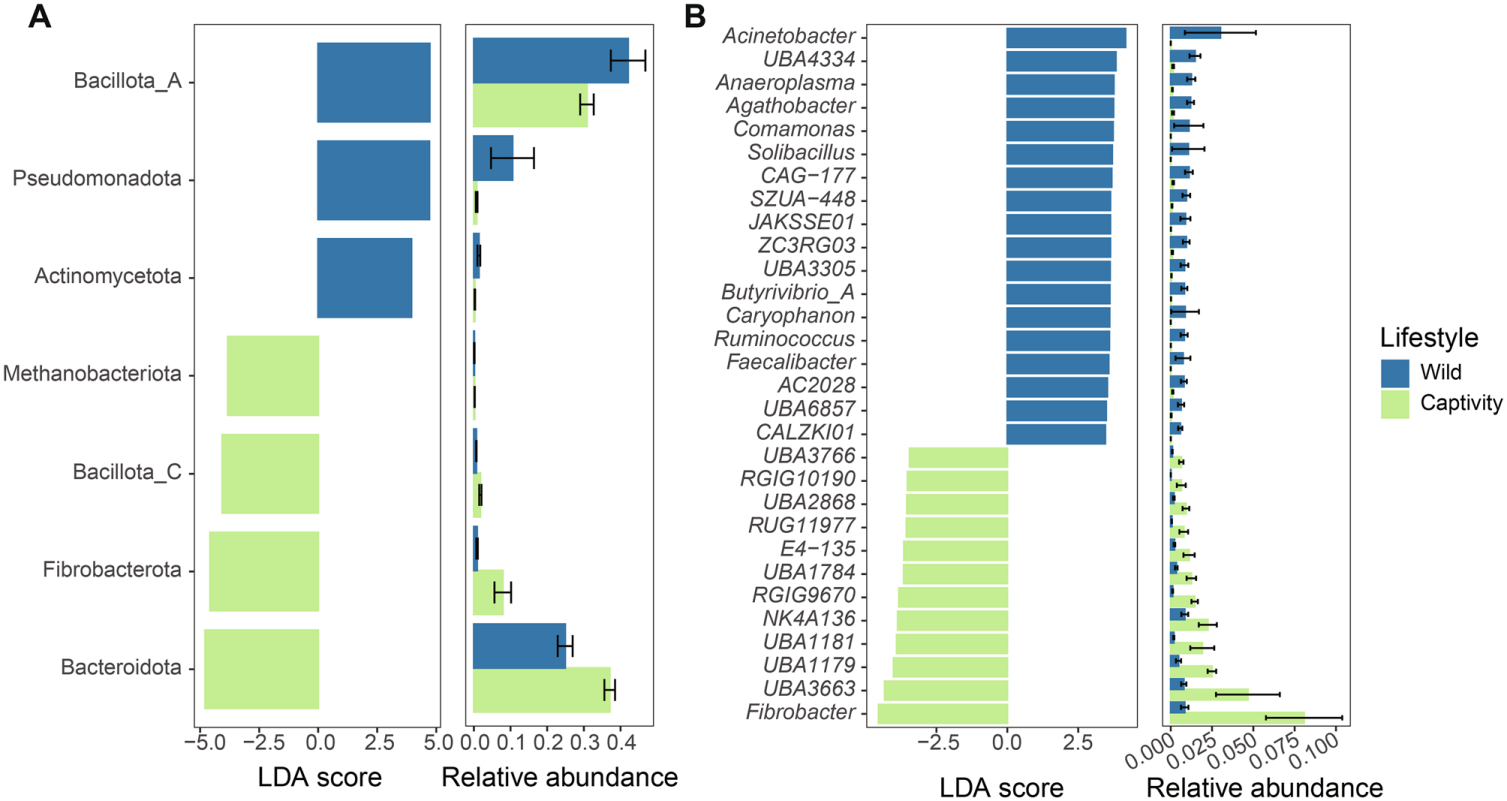
Effects of lifestyle on gut microbial composition in Asian elephants. Difference analysis of the species composition of Asian elephant gut microbes at the phylum level (**A**) and genus level (**B**) between elephants in the wild and captivity. The left bar graph shows the LDA score, where the color indicates the direction of enrichment, while the right bar graph represents the relative abundance of the species

We investigated the disparity in gut microbiota composition and function between wild (Mengyang and Jingne) and captive (WEV) Asian elephants. Our findings revealed that there were no significant differences in α diversity indices such as richness and the Shannon index (Fig. 3F-G). The PCoA plot demonstrated distinct clustering patterns between samples from different groups, indicating substantial variations in gut microbes between wild and captive Asian elephants (Fig. 3 H). To further examine the differences, we employed LEfSe analysis revealing that Bacillota_A, Pseudomonadota and Actinomycetota were significantly enriched taxa in the wild elephants, whereas Bacteroidota, Fibrobacterota, Bacillota_C and Methanobacterita were found to be enriched in captive elephants (Fig. 5A). At the genus level, several genera including *Acinetobacter*, *UBA 4334*, and *Comamonas* were found to be enriched in the wild elephants (Fig. 5B). Conversely, some genera associated with human activities such as *Fibrobacter*, *UBA3663*, and *UBA1179* exhibited enrichment in captive individuals (Fig. 5B).

Although significant differences were observed among microbial communities at the species level, variations in functional profiles were relatively limited. We observed that only one CAZyme (CE10) was enriched in the gut microbiota of captive elephants. In contrast, lipid metabolism pathways, elfamycin resistance, and two virulence factors (VF0461 and VF0350) were enriched in the wild lifestyle group. These findings suggest that the captive lifestyle had not substantially altered the functional composition of the intestinal microbiota.

## Discussion

In 2021, the north and south migrated Asia elephants held global headlines. The cause, the influence, and the conservation strategy related to this behavior were a matter of concern [24, 25]. Targeting the gut microbiota in wildlife research offers novel avenues for assessing wildlife health, understanding behavior, and evaluating impacts. The close association between the gut microbiota and host nutrition, health, genetic diversity, and adaptive evolution is widely acknowledged [52, 53]. In recent years, there has been a growing interest in applying metagenomic methods and technologies to address ecological, evolutionary, and conservation issues in wildlife research [54–56]. Despite several studies have demonstrated that the animal intestinal microbiota harbors a vast reservoir of unexplored microbial species, strains, genes, and functional clusters, the extent of our understanding regarding animal gut microbiomes, particularly in the realm of wild animals, remains limited. This knowledge gap also extends to Asian elephants - the largest terrestrial mammal on the Asian- which is classified as an endangered species (EN) by the International Union for Conservation of Nature (IUCN) in 1997. Our project provides detailed information of the taxonomic and functional diversity within the gut microbiota of Asian elephants, as well as factors affecting its composition.

The advancements in metagenomics sequencing technologies and the development of algorithms for assembly of MAGs have facilitated a comprehensive characterization of microbial community composition at the species level and provided a solid foundation for further investigations. Our project supplements and expands previous studies by performing a comprehensive survey on the gut microbial communities of Asia elephants, establishing comprehensive genome and gene catalogs. We envisaged that these catalogs will serve as a valuable basis for future analyses of the gut microbiota on Asian elephants. From the species annotation, over 93% of the SGBs represent potentially novel species. The findings indicate that despite extensive research on the diversity of gut microbiota in various animal species, there remains a significant number of unidentified microbial species within the wildlife microbiota needing further investigation. The SGBs generated in the current study clearly mitigate the problem of underrepresentation of data on the elephant gut microbiota in existing databases and greatly improved the average coverage of the high-quality reads. In addition, our study also contributes to information of the virulence and antibiotic resistance genes present in the gut microbiota of elephants. For example, the species *E. coli* and *K. electrica* exhibited the highest abundance of ARGs and may possess a potential for the acquisition and dissemination of ARGs in environmental settings. *E. coli* also harbors the highest number of virulence factors deserving attention in relation to wildlife conservation. The comprehensive investigation of these gut microbiota indicators and associated genes will directly contribute to the evaluation of the health status of Asian elephant populations and the enhancement of their habitats.

We found that captivity and migration influenced the composition and functional potential of the gut microbiota of Asian elephants. For the captive lifestyle, although significant differences were observed at the species level of gut microbiota, these changes had less impact on the functional composition of intestinal microbiota. This could potentially be attributed to similarities in geographical location, vegetation coverage, and dietary structure between elephants in captivity at WEV and their wild counterparts. We demonstrated disparities in gut microbiota between captive and wild herds, particularly at the species level, which have significant implications for designing feeding regimens, nutritional strategies, and health management protocols for captive populations, especially when addressing digestive ailments. For migration, the process of north-migration was accompanied by an increase in the diversity of the gut microbiota, increasing the abundance of Bacteroidota, and diminishing the presence of genera harboring potential pathogens indicating that the bacterial community structure of northward migrating elephants correlates with health, reflected by the good physical condition of this group. Further, the samples of north-migration provide a comprehensive representation of China’s wandering elephants. There are numerous hypotheses regarding the causes of migration in these wandering elephants, and no conclusion has been reached. Considering the historical wide distribution of Asian elephants and the fact that some wandering populations seems to harbor a gut microbiota associated with good health status, raise the question as to whether long-distance migration for more food and a specific gut microbiota environment represent a necessary condition for expansion of Asian elephant populations. Long-distance migration may also be accompanied by risks and uncertainties. The southward migration was accompanied by changes in the gut microbiota indicating an exposure to a less favorable environment including an increase in the capacity for biodegradation and metabolism of xenobiotics. During the southward migration, the elephant herd traversed regions proximal to human activities, such as rubber cultivation and fruit cultivation, where a portion of their dietary intake was linked to crops containing chemical residues. The alterations observed in the gut microbiota thus seemed intricately connected to the specific migratory events. Understanding how such events may affect the composition and functional potential of the gut microbiota may constitute a valuable research area of value for future conservation efforts. Hence, these findings not only enhance our insight into the Asian elephant’s intestinal microbiota, but more significantly, offer new research target for monitoring the health status of wildlife, conducting habitat assessments and habitat reserve construction.

The investigation of the gut microbiota in wild animals holds significant implications; however, obtaining high-quality samples for analysis remains a major challenge in wildlife research, especially when collecting fresh fecal samples from wild animals in their natural habitats. Dense forest coverage in the natural habitat of wild Asian elephants complicates the process of locating and tracking herds to obtain fresh elephant fecal samples. Despite these difficulties, a total of 82 fecal samples were collected, including 73 from wild Asian elephants, providing critical insights into their gut microbiota composition. Only 3 samples were collected in the Mengyang district during the designated sampling period, and only 2 were successfully sequenced for further analysis. In view of the fact that Mengyang is the original habitat for both south and north migrating elephants, even just two samples contribute valuable information. In order to mitigate the impact of a limited sample size, we conducted a comparative analysis on the variations in gut microbiota between northward and southward migrating elephants in this study. To analyze the difference between the wild and captive herds, the samples from Mengyang and Jingne were selected as representatives of the wild population and compared to the captive herd. Due to insufficient sample collection from other natural habitats besides Jingne, our current dataset is inadequate for comprehensively exploring patterns of wild elephant gut microbiota across different natural habitats and interactions between gut microbiota and the surrounding environment. Collecting additional samples from the same habitat during the same period and conducting longitudinal studies on elephant populations within the same habitat will enhance our understanding of these dynamics. This approach will facilitate a deeper characterization of the elephant gut microbiota and enhance conservation strategies through improved understanding of host-microbe-environment relationships.

## Electronic Supplementary Material

Below is the link to the electronic supplementary material.

Supplementary Material 1

Supplementary Material 2

Supplementary Material 3

## Data Availability

All the clean data, assembled contigs, and MAGs have been deposited into the CNGB Sequence Archive (CNSA) of China National GeneBank DataBase (CNGBdb) with accession number CNP0004215 at https://db.cngb.org/.
