# Supplementary figures and images for: Comprehensive catalog of gut microbial genomes in Asian elephants: insights from shotgun metagenomics
Source: Anim Microbiome. 2026 Mar 3;8:40. doi: 10.1186/s42523-026-00533-0 (PMC13064403; doi:10.1186/s42523-026-00533-0)

# Supplementary Figure 1

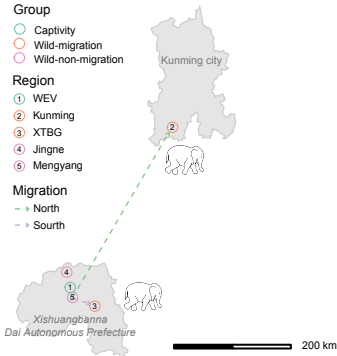

Supplement: Supplementary file 1 — Supplementary Material 1 [file 42523_2026_533_MOESM1_ESM.zip › 42523_2026_533_MOESM1_ESM/Supplementary Figure 1.pdf]

Supplementary Figure 2

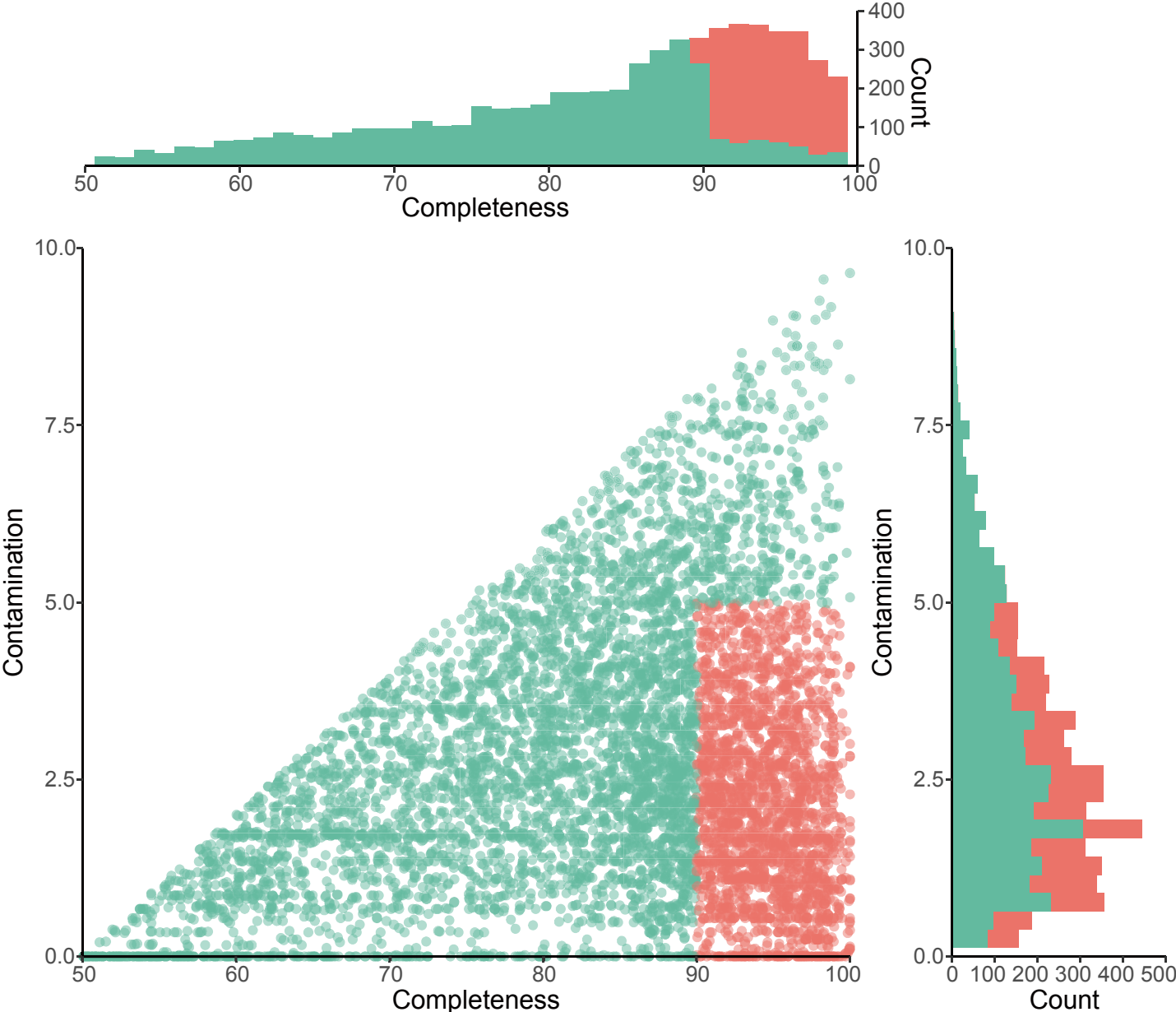

Supplement: Supplementary file 1 — Supplementary Material 1 [file 42523_2026_533_MOESM1_ESM.zip › 42523_2026_533_MOESM1_ESM/Supplementary Figure 2.pdf]

# Supplementary Figure 3

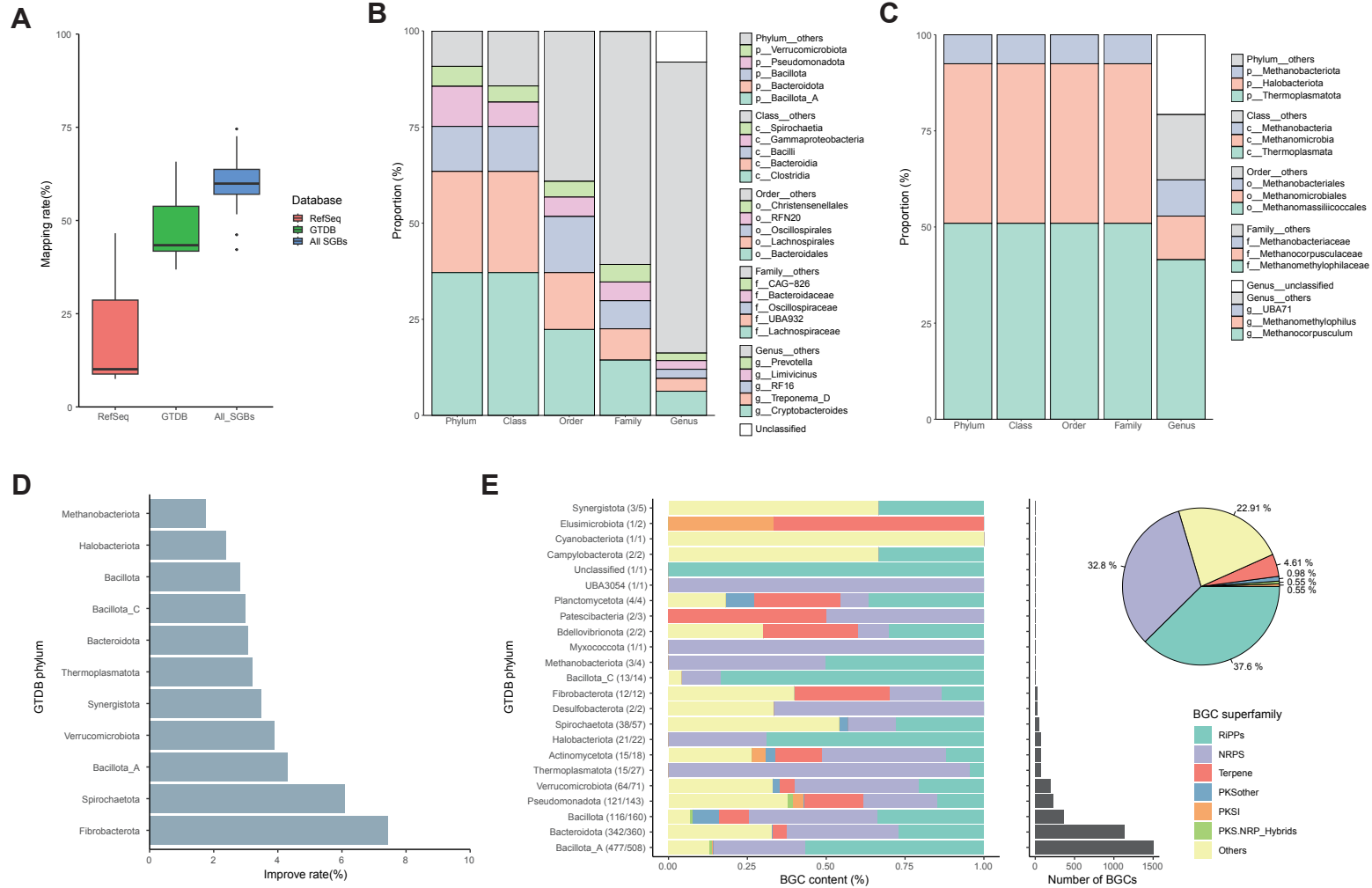

Supplement: Supplementary file 1 — Supplementary Material 1 [file 42523_2026_533_MOESM1_ESM.zip › 42523_2026_533_MOESM1_ESM/Supplementary Figure 3.pdf]

Supplementary Figure 4

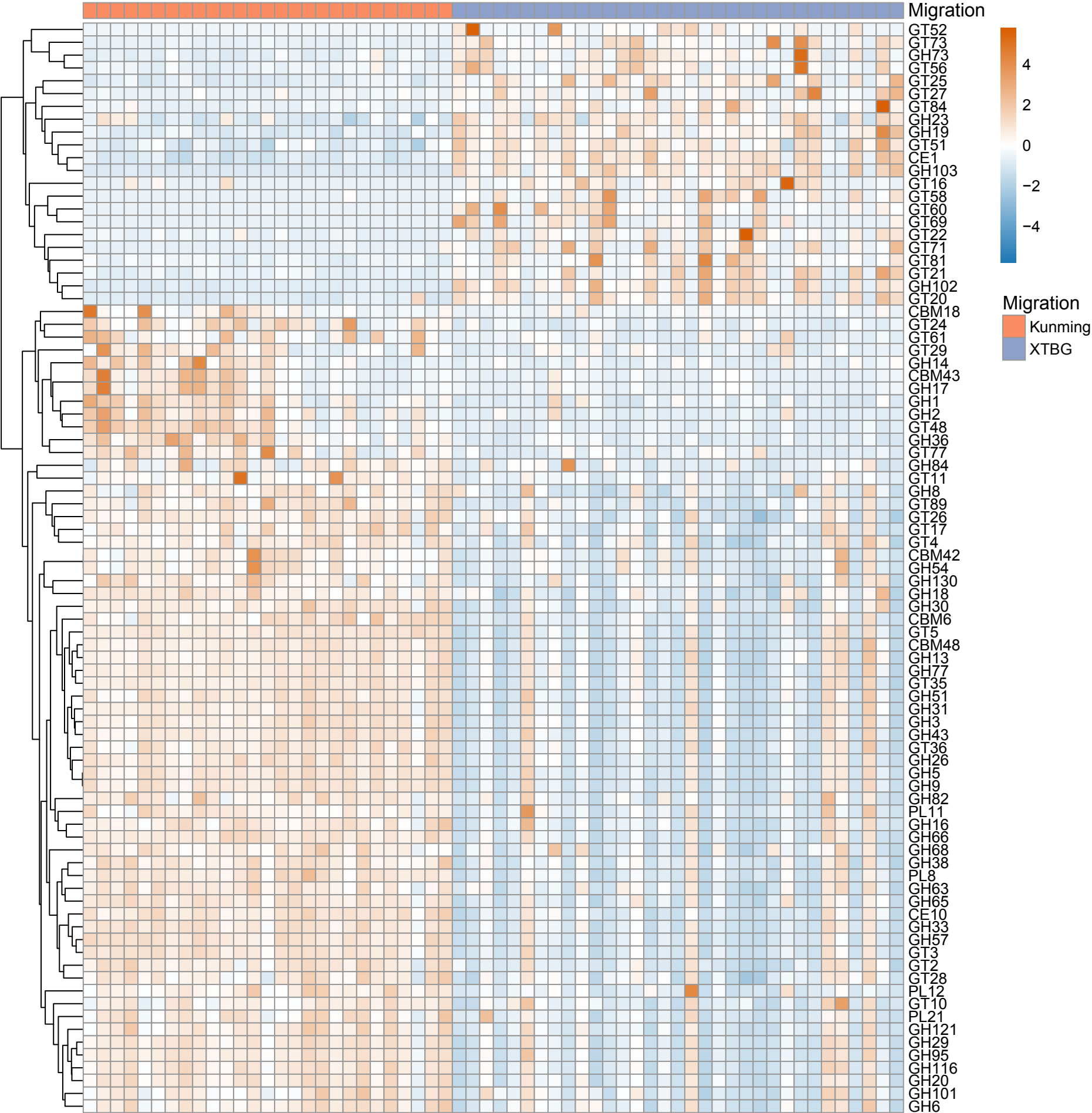

Supplement: Supplementary file 1 — Supplementary Material 1 [file 42523_2026_533_MOESM1_ESM.zip › 42523_2026_533_MOESM1_ESM/Supplementary Figure 4.pdf]

Supplementary Figure 5

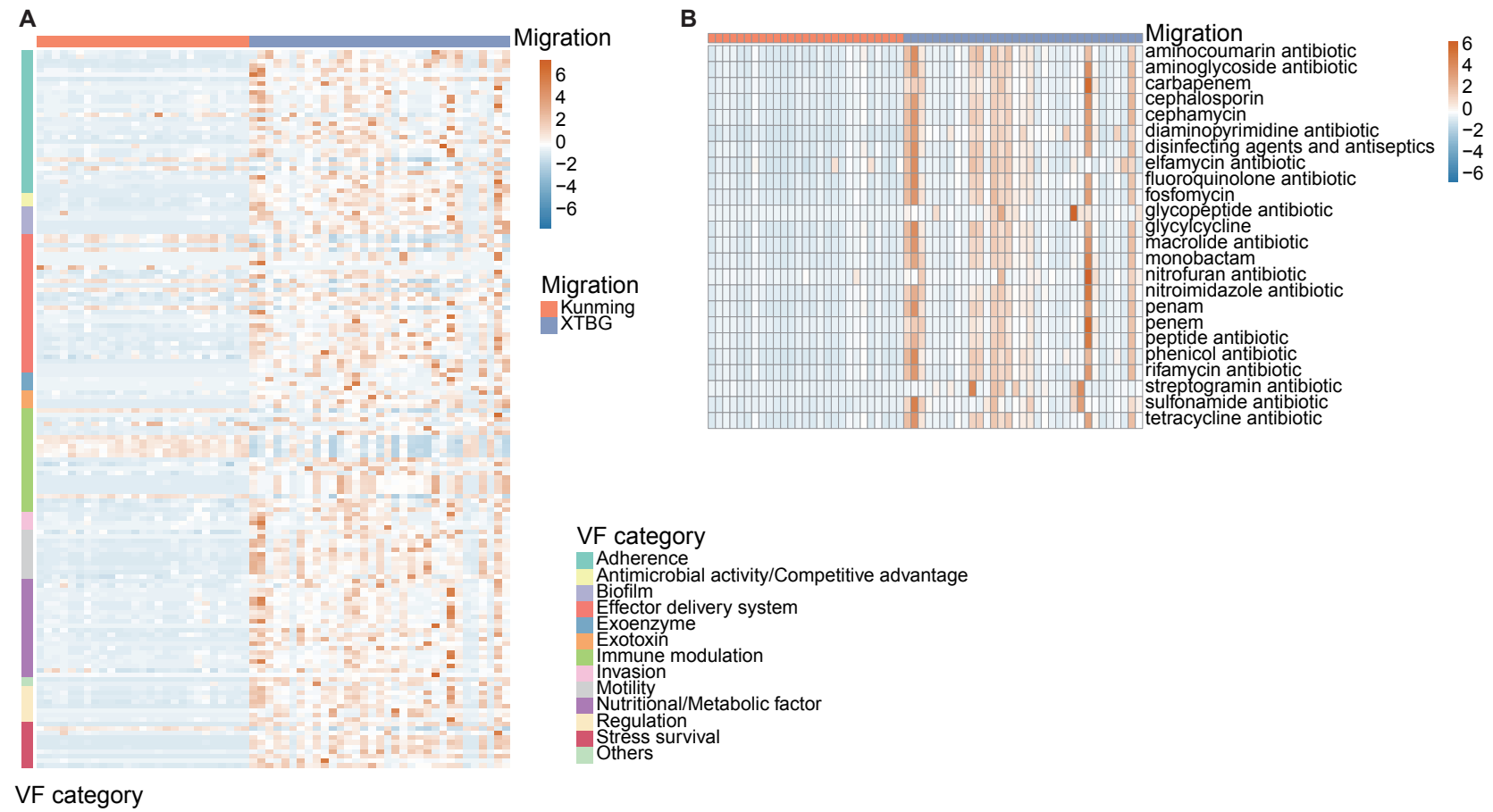

Supplement: Supplementary file 1 — Supplementary Material 1 [file 42523_2026_533_MOESM1_ESM.zip › 42523_2026_533_MOESM1_ESM/Supplementary Figure 5.pdf]
