# Supplementary material for: Comprehensive catalog of gut microbial genomes in Asian elephants: insights from shotgun metagenomics
Source: Anim Microbiome. 2026 Mar 3;8:40. doi: 10.1186/s42523-026-00533-0 (PMC13064403; doi:10.1186/s42523-026-00533-0)
Supplement: Supplementary file 3 — Supplementary Material 3 [file 42523_2026_533_MOESM3_ESM.docx]

## Additional information

**Additional file 1: Supplementary Figure 1. Sample distribution information.** A total of 82 samples were collected, distributed in five regions in the Yunnan Province, namely Kunming (27 samples), Mengyang (2 samples), Jingne (11 samples), the Wild Elephant Valley (WEV, 9 samples), and Xishuangbanna Tropical Botanical Garden (XTBG, 33 samples). The number represent different regions, and the colors represents different groups. The dotted line represents the migration route, and the color represents the migration direction. The north migration represents migration from Mengyang to Kunming, and the south migration represents migration from Mengyang to XTBG.

**Additional file 2: Supplementary Figure 2. Statistics of completeness and contamination of 6,313 MAGs.** Red plots represent high-quality MAGs, green plots represent medium-quality MAGs.

**Additional file 3: Supplementary Figure 3. Species composition and functional characterization of representative genomes of Asian elephant gut microbes.** (A) Mapping rates of high-quality reads from 82 samples against the 1,421 SGBs and two public databases (RefSeq and GTDB r214). (B) Taxonomy of bacteria in the 1,421 SGBs. Only the top five most frequently observed taxa are shown in the figure, the rest of the lineages are shown as ‘Others’. The white bars show the proportion of unclassified SGBs in each rank. (C) Classification of archaea in the 1,421 SGBs. Only the top three most frequently observed taxa are shown in the figure, the rest of the lineages are shown as ‘Others’. The white bars show the proportion of unclassified SGBs at each rank. (D) The improvement rate of unknown SGBs compared to GTDB r214 among the 1,421 SGBs at the phylum level. (E) The distribution of the seven BGC superfamilies in each phylum. The numbers in parentheses are the numbers of SGBs that harbor the predicted BGC superfamilies and the number of all SGBs in each phylum. The bar chart on the left is the proportion of the BGC superfamilies in each phylum. The bar chart on the right presents the number of BGCs, and the pie chart on the right presents the overall proportion of the BGC superfamilies.

**Additional file 4: Supplementary Figure 4. Differential enrichment of CAZymes between Kunming and XTBG.**

**Additional file 5: Supplementary Figure 5. Effects of migration on gut microbial function in Asian elephants.** (A) Differential enrichment of VF categories between Kunming and XTBG. (B) Differential enrichment of antibiotic resistance types between Kunming and XTBG.

**Additional file 6: Supplementary Table 1. Data summary of the samples.** (A) Detailed information for each sample. (B) Detailed data information for each sample.

**Additional file 7: Supplementary Table 2. Information of assembled MAGs.**

**Additional file 8: Supplementary Table 3. Summary of SGBs.**

**Additional file 9: Supplementary Table 4. The expanded diversity of species at the phylum level.**

**Additional file 10: Supplementary Table 5. Summary of BGCs annotated in all SGBs.**

**Additional file 11: Supplementary Table 6. Summary of ARGs annotated in all SGBs.**

**Additional file 12: Supplementary Table 7. Summary of VFs annotated in all SGBs.**

**Additional file 13: Supplementary Table 8. Summary of CAZymes annotated in all SGBs.**

**Additional file 14: Supplementary Table 9. Profiling of the 1,421 SGBs from the 82 samples.**
